# Genome-Wide Identification and Expression Profiling Analysis of SWEET Family Genes Involved in Fruit Development in Plum (*Prunus salicina* Lindl)

**DOI:** 10.3390/genes14091679

**Published:** 2023-08-25

**Authors:** Cuicui Jiang, Shaomin Zeng, Jun Yang, Xiaoan Wang

**Affiliations:** 1 Fruit Research Institute, Fujian Academy of Agricultural Sciences, Fuzhou 350013, China; zengshaomin1@163.com (S.Z.); y04116022@163.com (X.W.); 2 College of Food and Bioengineering, Bengbu University, Bengbu 233030, China; yj1904735520@sina.com

**Keywords:** sugar transporters, gene expression, plum, *PsSWEETs*, fruit development

## Abstract

SWEETs (sugars will eventually be exported transporters) play a vital role in longer-distance sugar transportation, and thus control carbon flow and energy metabolism in plants. *SWEET* genes have been identified in various plant species, but their functions in fruit development remain uncharacterized. Here, we isolated 15 putative *PsSWEETs* from the *Prunus salicina* genome. For further analysis, comprehensive bioinformatics methods were applied to determine the gene structure, chromosome distribution, phylogeny, cis-acting regulatory elements, and expression profiles of *PsSWEETs*. qRT-PCR analysis suggested that these *SWEETs* might have diverse functions in the development of plum fruit. The relative expression levels of *PsSWEET1* and *PsSWEET9* were obviously higher in ripened fruit than the ones in other developmental stages, suggesting their possible roles in the transport and accumulation of sugars in plum fruit. Positive correlations were found between the expression level of *PsSWEET3/10/13* and the content of sucrose, and the expression level of *PsSWEET2* and the content of fructose, respectively, during the development of ‘Furongli’ fruit, suggesting their possible roles in the accumulation of sucrose and fructose. The current study investigated the initial genomic characterization and expression patterns of the SWEET gene family in plum, which could provide a foundation for the further understanding of the functional analysis of the SWEET gene family.

## 1. Introduction

Sugars, the principal products of photosynthesis in higher plants, can be used as a significant source of carbon skeletons for the synthesis of various cellular compounds, osmolytes, signaling molecules, and transport molecules, as well as a temporary energy carrier [1,2,3]. In addition to providing energy to plants, sugars also serve as crucial components of metabolic intermediates that play important roles not just in plant growth, but also in abiotic and biotic stress responses [4,5,6]. The cellular exchange of sugars from cells or subcellular compartments requires the involvement of multiple transporters, such as monosaccharide transporters (MSTs), sucrose transporters (SUTs), and SWEETs [7,8,9,10]. Among them, SWEET transporter proteins are primarily pH-insensitive and facilitate bidirectional transmembrane sugar transportation along the concentration gradient [11]. SWEET transporter proteins are capable of selectively transporting monosaccharides or disaccharides across the plasma membrane or within cells, and are widely distributed in prokaryotes, plants, humans, and other animals [12,13].

It is now widely acknowledged that many *SWEET* genes have crucial functions in regulating plant growth, encompassing phloem loading, nectar secretion, pollen development, seed filling, and biotic and abiotic stress responses [8,14]. In *Arabidopsis thaliana*, the initially identified SWEET gene was *AtSWEET1*, which acted as a single glucose transporter and contributed to flower development by providing nutrients to the gametophyte [2,15]. Compared with the wild type, the overexpression strains of *AtSWEET4* have a higher plant height, while the mutant strain exhibits a shorter height, and lower contents of fructose and glucose in the leaves. It also has been reported that the overexpression of *AtSWEET4* enhanced tolerance to freezing and drought stresses [16]. Previously, AtSWEET11 and AtSWEET12, which are localized at the plasma membrane (PM) of phloem parenchyma cells, were responsible for sucrose phloem loading in pollen development. Mutations in *AtSWEET*11 and *AtSWEET*12 significantly retarded embryonic development [1]. The identification of *ZmSWEET4c* in maize and its ortholog *OsSWEET4* in rice as crucial genes for grain filling has been reported. Mutants of these two genes resulted in an impaired seed filling [17]. These findings highlighted the significance of *SWEET* genes in seed development. VvSWEET4, a glucose transporter situated on the plasma membrane in *Vitis vinifera*, participated in the host interaction with *Botrytis cinerea* [18]. Several reports indicated that the SWEET gene family also plays a vital role in fruit development. For example, *SlSWEET12C* and *SlSWEET14* exhibit a relatively high expression in ripening the tomato fruit [19]. In banana, it was discovered that over 80% of the *MaSWEET*s genes were expressed throughout five developmental and ripening stages. Additionally, more than 27% of these genes exhibited high expression levels (FPKM > 10) at each stage [20]. The transcript levels of ten *MdSWEET*s were detected in apple leaves and fruit. The expression of *MdSWEET1.1/2* was twice as high in the fruit compared to the leaves and shoot tips, implying that its expression level might not be influenced by the concentrations of glucose, fructose, and sucrose in apple cells [21]. Taken together, the SWEET gene family has been found to participate in multiple vital biological processes in plants, including the growth, development of seeds, fruit, and pollen, environmental adaptation, and host–pathogen interactions.

Plum, a perennial deciduous fruit tree belonging to the *Prunus* genus in the *Rosaceae* family, is cultivated globally and considered an important traditional fruit tree [22]. Plum has a long history of cultivation and possesses strong adaptability [23]. It has been cultivated worldwide and has abundant genetic resources. To date, there are 30–40 species of *Prunus* plants in the world [24]. Over 800 varieties were found in China [25]. Among them, the ‘Furongli’ plum (*P. salicina* Lindl.), which has a red peel and red pulp, has been grown for over 700 years in Fujian Province, China [26]. It is considered to be an economically important fruit tree in China due to its beautiful appearance, beneficial antioxidant potential, and bioactive compounds [27]. Despite detailed investigations of the sugar, organic acid, and secondary compound contents in ‘Furongli’ plum through previous studies [28], there is still a lack of full understanding regarding sugar transport during the development of the ‘Furongli’ plum. In the current study, we conducted a comprehensive analysis of gene structures, conserved domains, expression patterns, and phylogenetic relationships of the SWEET gene family based on a whole-genome-wide level in plum. This comprehensive study serves to facilitate our understanding of the evolutionary patterns and the expression of *PsSWEETs* in plum fruit development.

## 2. Materials and Methods 

### 2.1. Identification and Phylogenetic Analysis of PsSWEET Genes

The genome sequences of *P. salicina* was downloaded from the Rosaceae Genome Database (https://www.rosaceae.org/, downloaded on 22 December 2022) [29]. *AtSWEET* genome sequences were downloaded from TAIR (https://www.arabidopsis.org/, downloaded on 22 December 2022). Firstly, the *Arabidopsis AtSWEET* genes was employed as a query sequence to perform a Blast search against the plum genome using the BLASTP tool with an E-value threshold of 1.0. Secondly, the HMMER3 software suite (version 3.3.2) was utilized to obtain the seed alignment file for the MtN3/saliva domain (PF03083) from the Pfam database (https://pfam.xfam.org/, downloaded on 25 December 2022) [30]. Subsequently, the HMMER3 software suite (version 3.3.2) was used to conduct HMM searches against the local protein databases of plum with an E-value < 1.0 [31]. Finally, the sequences of PsSWEET proteins were employed for the computation of basic biochemical characteristics using the ExPASy tool (http://www.expasy.org/tools, downloaded on 28 December 2022) [32], including molecular weight, theoretical isoelectric point, and hydrophilic mean. MEGA-X (v10.2.6) was used to investigate the phylogenetic and molecular evolutionary genetics of PsSWEET proteins with the default settings [33]. The neighbor-joining (NJ) approach was employed to construct the evolutionary tree with a bootstrap of 1000. The tree was further edited using iTOL (https://itol.embl.de, accessed on 29 December 2022) [34].

### 2.2. Analysis of Gene Structure, Protein Motif, and Promoter Region 

The gene structures of *PsSWEETs* were virtualized using the Gene Structure Display Service (available online: http://gsds.cbi.pku.edu.cn, accessed on 20 February 2023) [35]. In addition, the conserved motifs were displayed by using the online tool MEME Suite 5.1.1 (http://meme-suite.org/, visited on 20 February 2023) [36]. The *cis*-regulatory elements from gene promoters (1500 bp before ATG) were analyzed using the PLACE database (http://www.dna.affrc.go.jp/PLACE/, visited on 25 February 2023).

### 2.3. Gene Duplication, Synteny, and Chromosomal Locations Analysis of PsSWEET Genes 

Synteny analysis among the SWEET family members was carried out using MCScanX and Tbtools [37,38]. Additionally, the synteny analysis of plum with *Arabidopsis*, apple, pear, and prune was also constructed using the same tools. The positions of the SWEET genes on chromosomes were drawn using MapInspect software (version 1.0).

### 2.4. Total RNA Isolation and Quantitative Real-Time PCR Analysis (qRT-PCR) 

Plant material was collected as previously described [39]. Briefly, the fruits of the ‘Furongli’ plum were harvested from 6-year-old field-grown trees in Fuda Village, Fujian Province, China. Fruit samples were picked 23, 43, 70, 98, 112, 127, and 157 days after flowering (DAF), respectively. All the trees received the standard horticultural practices and insect prevention. The fruit samples were stored at −80 °C until use. After that, they were peeled and sliced into appropriate pieces and frozen in liquid nitrogen immediately. For qRT-PCR analysis, the total RNA was isolated using the RNA prep Pure Plant Total RNA Extraction Kit (Tiangen, Beijing, China) following the manufacturer’s instructions. First-strand cDNA was synthesized for qPCR using the PrimeScript RT reagent kit with gDNA Eraser (Takara, Dalian, China). The qRT-PCR reactions were conducted in 20 µL volumes, utilizing 1 µL of cDNA and 2 × SYBR Premix Ex TaqTM II (Tli RNaseH Plus, TaKaRa), based on the Eppendorf RealPlex4 system (Hamburg, Germany). The experiment procedures of qRT-PCR were 40 cycles of 95 °C for 15 s and then 68 °C for 30 s. The actin gene was utilized as the control standard, and the 2^−ΔΔCT^ method was employed to analyze the relative expression levels of the genes [40]. Three biological and three technical replicates were performed. The primer sequences are provided in Table S1.

### 2.5. Statistical Analysis

The statistical analysis of the qRT-PCR data was performed via one-way ANOVA using SPSS19 software. The means were separated using Duncan’s new multiple-range test *p* < 0.05. Different letters were used to indicate significant differences (*p* < 0.05). The *ggcor* package was used to perform the correlation test in the R circumstance [41]. 

## 3. Results

### 3.1. Identification and Phylogenetic Analysis of PsSWEET Family Members

After confirming the SWEET domain using the CDD and PFAM (PF03083) databases, a total of 15 *PsSWEET*s were discovered and named based on their corresponding orthologous genes in *A. thaliana* (Table 1). The molecular characteristics of these proteins are described in this study. These proteins varied in sizes ranging from 134 (in the case of PsSWEET6) to 291 (in the case of PsSWEET9), and also varied in isoelectric points ranging from 6.27 to 10.44 (Table 1). The PsSWEET proteins displayed a high degree of variability in their structure, implying that these proteins may have different roles in various biological processes or under different growth conditions.

**Figure 1:**
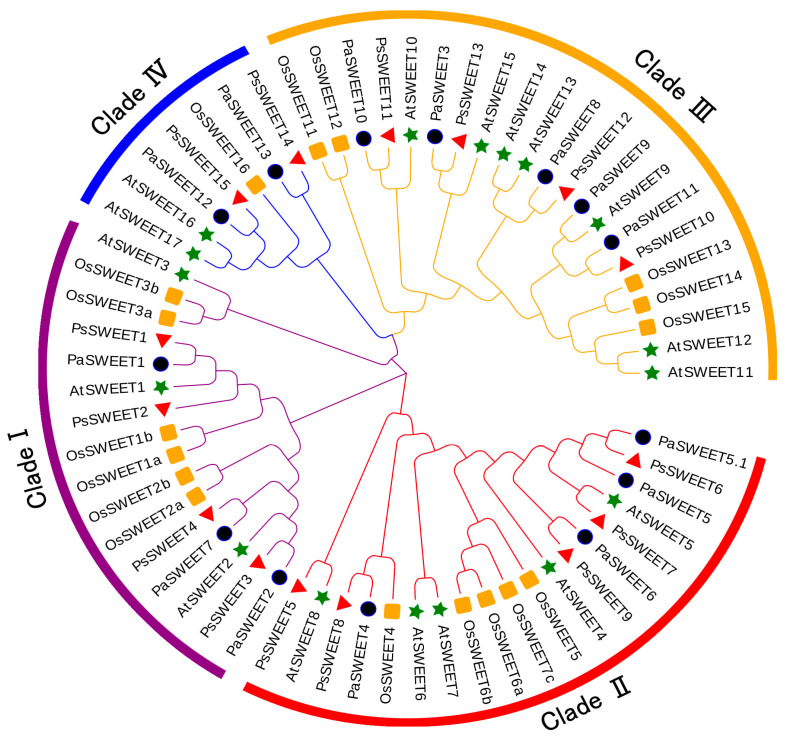
Phylogenetic analysis of SWEET proteins from *Prunus* s*alicina*, *Prunus avium*, *Oryza sativa*, and *Arabidopsis thaliana*. The SWEETs from *P.* s*alicina*, *P. avium*, *O. sativa*, and *A. thaliana* were marked with a red triangle, purple circle, yellow square, and green star, respectively. The phylogenetic tree of the SWEET family protein sequences was conducted using MEGA 6.0 via the neighbor-joining method with 1000 bootstrap replicates. The gene family was divided into four SWEET subfamilies, clade I, II, III, and IV.

To investigate the evolutionary events of the SWEET gene family in *P. salicina*, we constructed a phylogenetic tree based on *A. thaliana*, *Oryza sativa*, and *Prunus avium*. All PsSWEETs proteins were successfully divided into four clades (I–IV) according to the phylogenetic tree (Figure 1). Among the four clades (I–IV), clade III was the largest clade with 21 members, containing four PsSWEET, six AtSWEET, five OsSWEETs, and six PaSWEETs members. Clade I had the same PsSWEET members (Figure 1). Clade IV was the smallest clade with seven members, including two PsSWEETs, two AtSWEETs, one OsSWEETs, and two PaSWEETs members. The distribution of PsSWEETs, AtSWEETs, OsSWEETs, and PaSWEETs proteins encompassed all clades. Notably, PsSWEETs exhibited a closer relationship with PaSWEETs compared to AtSWEETs and OsSWEETs (Figure 1).

### 3.2. Analysis of Gene Structure, Motif, and Cis-Element in Promoter Regions of PsSWEETs

**Figure 2:**
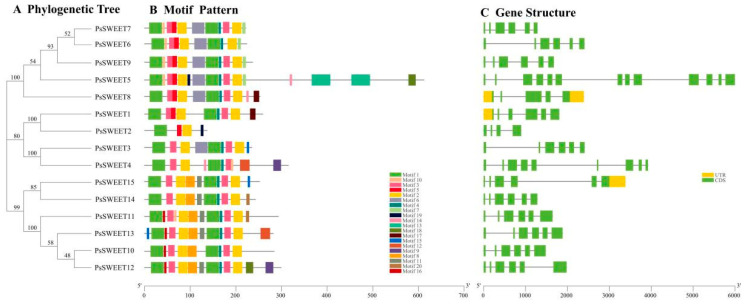
Phylogeny, motifs, and exon–intron structures of PsSWEET proteins or genes. (**A**) Neighbor-joining phylogenetic tree of PsSWEETs. (**B**) Distribution of the conserved motifs in PsSWEET proteins. Twenty conserved motifs are marked with different colored boxes. (**C**) Exon-intron structure of *PsSWEETs* genes. Green boxes indicate exons, yellow boxes indicate UTR, and black lines indicate introns, respectively.

Generally speaking, the 15 PsSWEET proteins were clustered into three categories (Figure 2A). The 20 conserved motifs (motif 1–motif 20) of the *PsSWEET* gene family members were predicted using the MEME program. Motifs 1, 2, 5, and 19 were found in all PsSWEETs (Figure 2B). PsSWEET5 had the largest number of motifs, containing 16 motifs, while PsSWEET2 had the fewest, only containing 2 motifs. These results suggest that the majority of PsSWEETs grouped together share similar conserved motifs, which provides additional support for the phylogenetic results. The exon/intron structures of the *PsSWEET* gene family members were analyzed. Among the SWEET genes in plum, five introns were found in most *PsSWEETs* (Figure 2C). *PsSWEET5* has the most prominent intron numbers, containing 12 introns. Compared to other *PsSWEET* genes, *PsSWWET2* possesses the lowest number of introns, containing three introns (Figure 2C). The intron numbers of *PsSWEETs* ranged from three to twelve, exhibiting considerable variation. Remarkably, the *PsSWEETs* contained 5 exons in Clades III and IV, 3–8 exons in Clade I, and 4–12 exons in Clade II (Figure 2C). The exon–intron structure of the most of *PsSWEETs* within the same groups displayed a similar exon–intron structure, suggesting a high level of conservation. 

**Figure 3:**
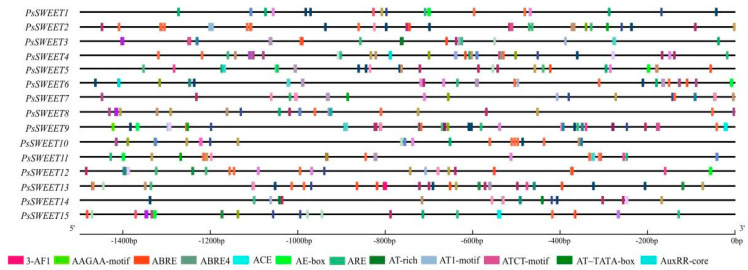
Predicted cis elements in the promoter regions of *PsSWEET* genes, which were presented as colored rectangles. The numbers on the top indicate the relative positions to the start codon.

To enhance the comprehension of *PsSWEETs*’ transcriptional regulation and potential functions, 5′ regulatory elements in the 1500 bp sequences located upstream of their translation start sites were analyzed using the PlantCARE server. Twelve different types of putative cis-elements responsive to biotic stresses, fruit development, and hormones, including 3-AF1, AAGAA-motif, ABRE, AE-box, ARE, AT-rich, AT1-motif, AT~TATA-box, AuxRR-core, were found in the 1500 bp promoters of *PsSWEET* genes (Figure 3). 

### 3.3. Duplication and Synteny Analysis of PsSWEET Family Members

**Figure 4:**
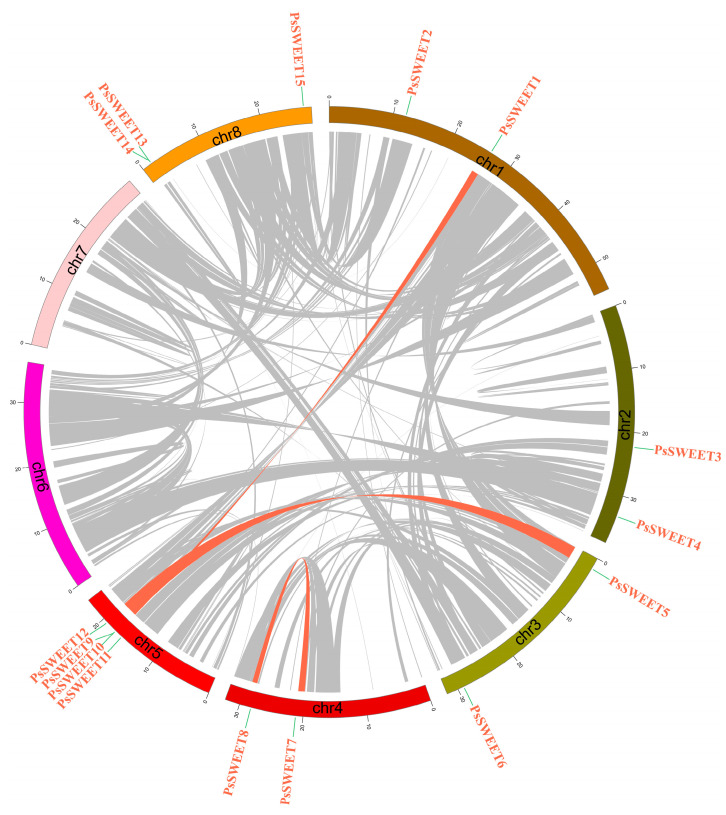
Gene duplication analysis of *PsSWEET* genes. Pink lines connect the syntenic regions between plum *PsSWEET* genes.

Previous studies have shown that gene duplication, including tandem and segmental duplication, plays a significant role in the evolution of plant genomes. Three segmental duplication events (*PsSWEET7/8*, *PsSWEET1/12,* and *PsSWEET5/9*) and two tandem duplication events (*PsSWEET9/10* and *PsSWEET13/14*) were identified in the present study (Figure 4). These results indicated that some *PsSWEET* genes were probably generated via gene segment or tandem duplication. 

**Figure 5:**
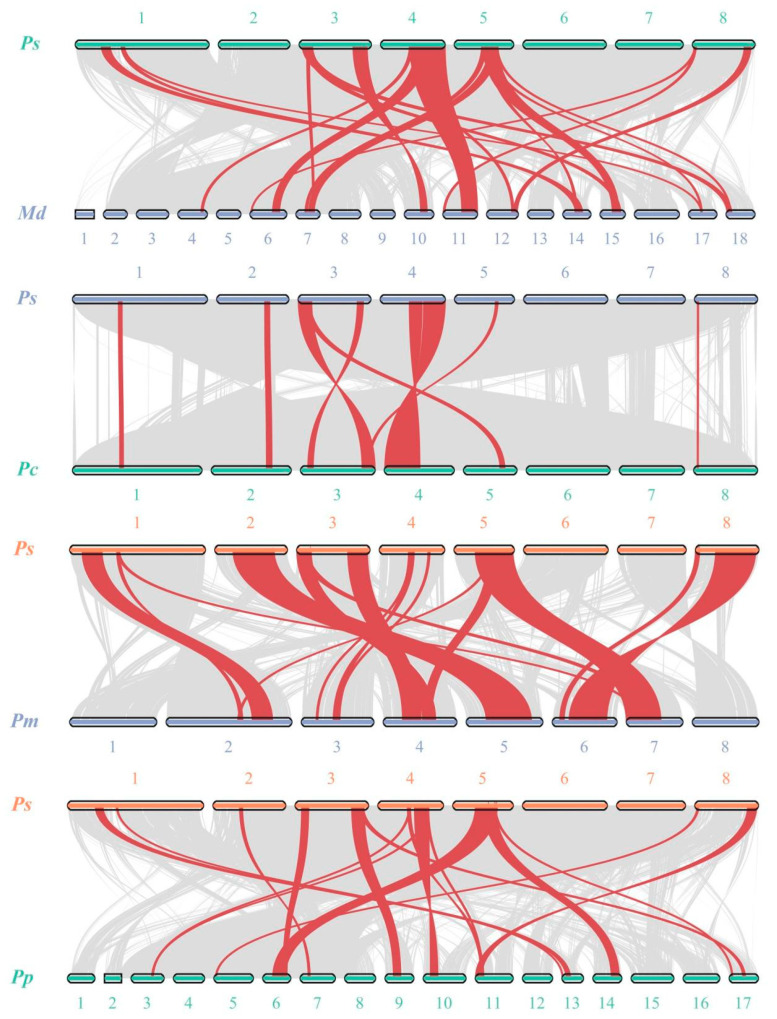
Synteny analysis of PsSWEET genes between *P. avium* and *Malus domestica*, *Prunus persica*, *Pyrus communis*, *Prunus mume*.

To explore the potential evolutionary clues of the *PsSWEET* gene family, a series of comparative syntenic maps of *P. avium* associated with the four *Rosaceae* plant species, including *Malus domestica*, *Prunus persica*, *Pyrus communis* and *Prunus mume*, were constructed in this study. The syntenic analysis of *PsSWEET* family members exhibited that 15 *PsSWEET* genes were in synteny with *M. domestica* (8), *P. persica* (10), *P. communis* (8), and *P. mume* (10) (Figure 5). 

### 3.4. Chromosomal Localization Analysis of PsSWEETs

**Figure 6:**
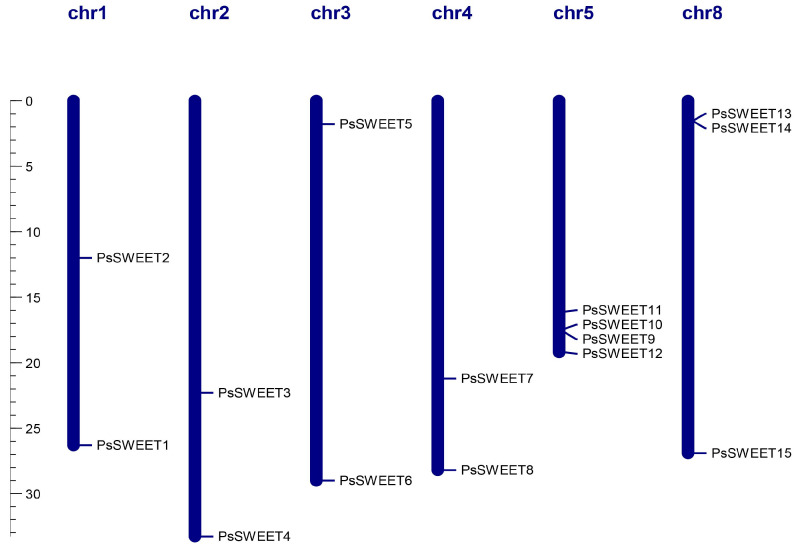
Schematic representations of the chromosomal location of the *PsSWEET* genes. The chromosome number is marked on each chromosome.

As shown in Figure 6, the distribution of all *PsSWEET* genes was uneven across the six LGs in the plum genome. Among these genes, there was no detection of *PsSWEET* genes on chromosomes 6 and 7. The *PsSWEET1/2* genes are observed on Chromosome 1, *PsSWEET3/4* are on Chromosome 2, *PsSWEET5/6* are on Chromosome 3, *PsSWEET7/8* are on Chromosome 4, and *PsSWEET9/10/11/12* are on Chromosome 8. Chromosome 5 has the largest number of *PsSWEET9* genes (*PsSWEET9/10/11/12*).

### 3.5. Verification of Key PsSWEET Family Members during Fruit Development via qRT-PCR 

**Figure 7:**
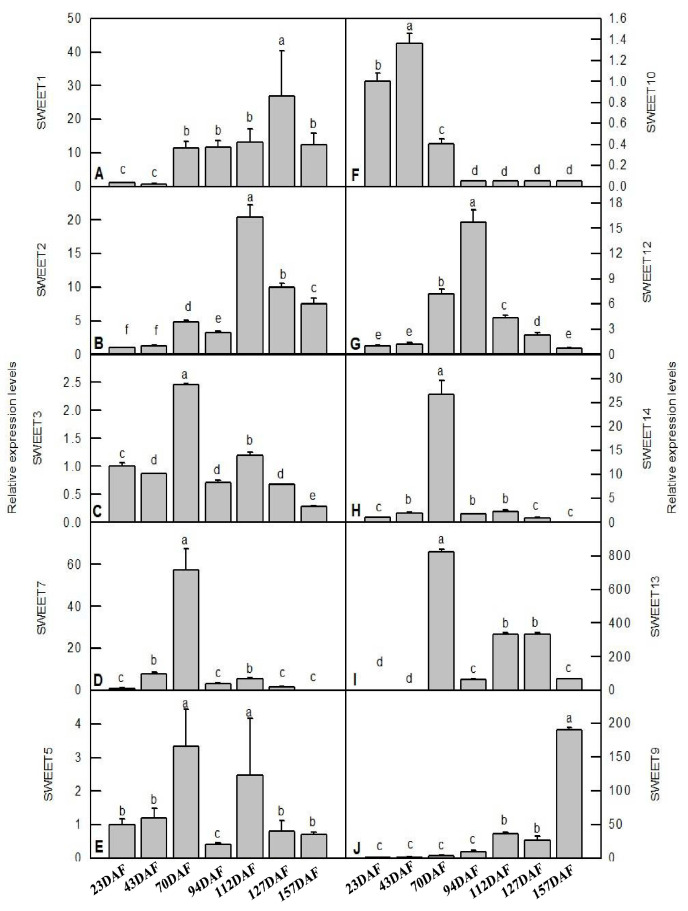
qRT-PCR analysis of key *PsSWEET*s during fruit development and ripening of ‘Furongli’ plum. (**A**) *SWEET1*; (**B**) *SWEET2*; (**C**) *SWEET3*; (**D**) *SWEET7*; (**E**) *SWEET5*; (**F**) *SWEET10*; (**G**) *SWEET12*; (**H**) *SWEET14*; (**I**) *SWEET13*; (**J**) *SWEET9*. Data are the means ± SD of three biological replications (n = 3). Different letters above the bars indicate a significant difference at *p* < 0.05.

The regulation of sugar transport from source to sink cells is a key factor in plant fruit development, and *SWEETs* are integral to this process by controlling sugar efflux transport [2]. In order to better understand the functions of *SWEETs* in ‘Furongli’ plums during fruit development and ripening, the expression levels of ten detectable *PsSWEETs* were determined using qRT-PCR (Figure 7). *PsSWEET1/9* showed relatively lower levels of expression at the early stages of fruit development, while it sharply increased at the later stages. Noteworthy is that the expression patterns of *PsSWEET3*, *PsSWEET4*, *PsSWEET7*, and *PsSWEET13* were similar during fruit development, and were the highest at 70 DAF. *PsSWEET5* had the highest expression level at 70 DAF and 112 DAF, and *PsSWEET14* had the highest expression level at 70 DAF. The expression levels of two other genes (*PsSWEET2* and *PsSWEET12*) peaked at 94 DAF, then decreased gradually. It is worth noting that the most of *PsSWEET* genes displayed higher expression at the early stage of fruit development compared to the late stage (Figure 7). 

### 3.6. Expression Patterns Analysis of PsSWEET Family Members during Fruit Development

**Figure 8:**
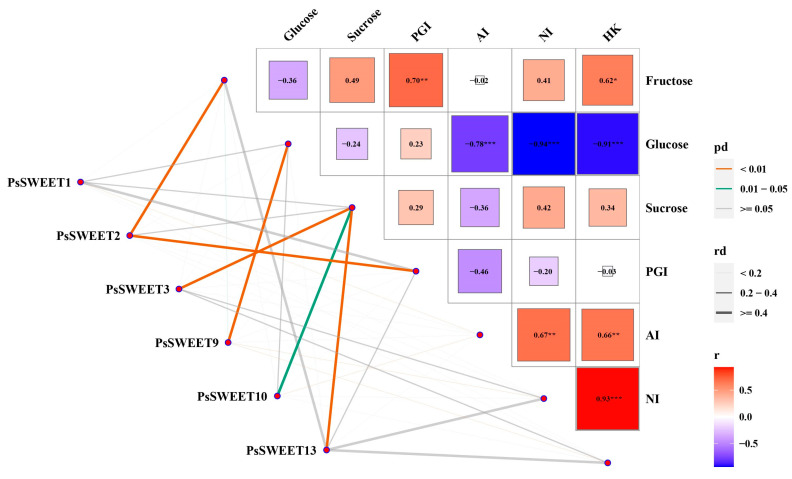
The correlations among the expression levels of the six key *PsSWEET* genes and soluble sugar contents (sucrose, fructose, and glucose), and related enzyme activity (PGI, HK, and AI). Data of soluble sugar contents and enzyme activities used for the correlation analysis were extracted from our previous reported literature [39]. The lines correspond to the expression correlations between the six key *PsSWEET* genes and the soluble sugar contents, and related enzyme activity. The colors of the lines represent the Mantel’s pd, and the thicknesses of the lines represent the percentage of the Mantel’s rd. The intensity of the color from blue to red and size of the box indicate the degree of the expression correlation among the soluble sugar metabolic. “*”, “**”, and “***” mean a significant difference at *p* < 0.05, 0.01, and 0.001, respectively.

A correlation analysis between the expression levels of the five key *PsSWEET*s and soluble sugar contents (sucrose, fructose, and glucose) and related enzyme activity (PGI, HK, NI and AI) during plum development was conducted [39]. The results showed that among the five key *PsSWEET* genes, except for *PsSWEET1*, the expression levels of each of the other genes were significantly positively correlated with soluble sugar contents, and related to enzyme activity in the sugar metabolic pathway (Figure 8). A significant positive correlation was observed between *PsSWEET9* and the content of glucose during the development of the ‘Furongli’ fruit. Furthermore, a significant positive correlation was found between *PsSWEET3/10/13* and the content of sucrose, respectively. A significant positive correlation was also found between *PsSWEET2* and PGI or the content of fructose, respectively (Mantel’s 0.01 < *p* < 0.05). Such a result might implicate the possible role of PsSWEET genes in the regulation of soluble sugar contents during the development of plum fruit.

## 4. Discussion

The plum, as one of the most essential fruits, has been cultivated on a global scale. It is rich in bioactive substances such as vitamin C, carotenoids, polyphenols, and anthocyanins [28,42,43,44]. Except for bioactive substances, sugars play a crucial role in determining the quality of fruits and also contribute to their caloric value [45]. Sucrose and hexoses (mostly glucose and fructose), which are commonly found in fruits, are the primary sugars. Sucrose is transported by the apoplastic pathway and unloaded from the phloem into the loquat and cucumber fruits. A previous study indicated that hexose absorbed from the fruit apoplasm into the fruit storage parenchyma cells was facilitated by the CsHT3, which acts as a hexose transporter [46]. Still, the process remains unclear. Recently, the SWEET family, a novel type of sugar transporter, may play important roles in regulating the unloading process of sugars. SWEET proteins have been found in many plant species, such as mulberry [47], wheat [48], *Arabidopsis* [49], tea [50], *Sorghum* [51], and physic nut [52]. However, only a few species have been used to elucidate the genomics and function of *SWEET* genes until now. From this point, we investigated the main molecular characteristics and expression profiles of *PsSWEET* gene family members in the current study. 

Here, 15 *PsSWEET* genes were identified based on the genome sequence of *P. salicina*. Their phylogenetic evolutionary relationship showed that these genes were classified into four subgroups (Figure 1). This classification is consistent with previous reports in banana [20], strawberry [53], tomato [54], soybean, watermelon [55], cabbage [56], and cucumber [57]. The majority of the *PsSWEET* genes grouped together share similar conserved motifs and gene structure, which provides additional support for the phylogenetic results (Figure 2). Similar results have been reported in other Rosaceae species, such as apple [58], pear [59], and loquat [47]. In terms of the phylogenetic relationship, gene structure, and conserved motifs, we found that the *PsSWEET* gene family might be relatively conservative in the process of evolution. It is widely recognized that gene duplication plays a significant role in the driving force for the expansion of gene families and greatly increases functional diversity [60]. The segmental and tandem gene duplications are considered as two key factors responsible for the generation and maintenance of gene families [61]. Three segmental duplication events (*PsSWEET7/8*, *PsSWEET1/12*, and *PsSWEET5/9*) and two tandem duplication events (*PsSWEET9/10* and *PsSWEET13/14*) were identified in the present study (Figure 4). Interestingly, the tandem duplication events (*PsSWEET9/10* and *PsSWEET13/14*) have divergent expression patterns during the development of plum fruits, which might be due to the different cis elements identified in these *SWEET* genes (Figure 3 and Figure 4). Therefore, the further details of transcriptional regulation need to be studied in the future. Only one segmental duplication event (*LcSWEET4* and *LcSWEET5*) and three tandem duplication events (*LcSWEET3a/3b*, *LcSWEET9a/9b*, and *LcSEET10/12*) were found in *Litchi chinensis* [62]. The varied number of *SWEET* genes in different plant species could be a result of evolutionary mechanisms, such as tandem or segmental duplication. Together, it is proposed that the expansion of *PsSWEETs* might play a considerable role in the various gene functions of *SWEET*.

In recent years, growing evidence indicates that SWEET proteins exhibit a broad distribution across various plant species and participate in diverse physiological roles, including plant growth, fruit development, and stress responses. As previously mentioned, many researchers have found that the SWEET family is involved in the fruit development process in many plant species, such as *M. domestica* [58], *Solanum lycopersicum* [63], and *V. vinifera* [18]. However, it has still remained unclear whether *PsSWEETs* in plum also play a role in fruit development. Further, higher expression levels for the *PsSWEET* gene were detected during the development of plum fruit via qRT-PCR. The results indicated that the SWEET family in plum has participated in the fruit development process, which is similar to strawberry. Some *PsSWEET* genes (*PsSWEET3/7/10/13/14*) with a higher expression were observed during the early stage of fruit development (Figure 7). In prior studies, the sucrose content of ‘Furongli’ increased at the early stage of fruit development and then gradually decreased [39]. We found that there was positive correlation between *PsSWEET3/10/13* gene expression and sucrose content during ‘Furongli’ development (Figure 8), which suggests that they play vital roles in the cellular transportation and unloading of sugars into fruits for sink storage. That is consistent with previous reports by Miao et al. [20]. Furthermore, *AtSWEET16/17*, a facilitative transporter, was demonstrated to mediate fructose transport across the tonoplast of roots and leaves in *Arabidopsis* [64,65,66,67]. Here, we also found that *PsSWEET2* has a positive correlation with the fructose level during fruit development, and *PsSWEET9* has a positive correlation with glucose levels during fruit development (Figure 8). These results indicate that *PsSWEET* genes have the potential to transport not just sucrose and glucose, but also fructose, which is supported by previous findings. Together, these data will supply abundant information for future investigations of *SWEET* gene functions for sugar transporters during fruit development.

## 5. Conclusions 

It is well known that many *SWEET* genes have crucial functions in regulating sugar transportation during fruit development. Here, we successfully investigated the gene structures, conserved domains, expression patterns, and phylogenetic relationships of the SWEET gene family in plum. Fifteen *PsSWEET* genes were identified in *P. salicina*. Our qRT-PCR analyses have shown that *PsSWEE2, PsSWEET3*, *PsSWEET4*, *PsSWEET5*, *PsSWEET7*, *PsSWEET13*, and *PsSWEET14* exhibited a relatively higher expression at the early stage of fruit development than at the later developmental stages. However, *PsSWEET1/9* showed relatively lower levels of expression at the early stage of fruit development, while it sharply increased in the late stages. The divergent expression patterns of some tandem duplication events, such as *PsSWEET9/10* and *PsSWEET13/14* during the development of plum fruits, might be due to the different cis elements identified in these SWEET genes, respectively. Positive correlations were found between the expression level of *PsSWEET3/10/13* and the content of sucrose, the expression level of *PsSWEET2* and the content of fructose, and the expression level of *PsSWEET9* and the content of glucose, respectively, during the development of the ‘Furongli’ fruit. Therefore, we proposed that *PsSWEET*s might have the potential to transport not just sucrose and glucose, but also fructose during ‘Furongli’ fruit development. 

## Figures and Tables

**Table 1:** Detailed information on 15 *PsSWEET* genes in plum.

Gene Name	Genome ID	Chr	Protein Length (aa)	Molecular Weight (kDa)	PI
EVM0028303	*PsSWEET1*	Chr1	283	31,379.05	8.43
EVM0026528	*PsSWEET2*	Chr1	253	27,767.83	8.19
EVM0011805	*PsSWEET3*	Chr2	244	26,849.05	9.45
EVM0012056	*PsSWEET4*	Chr2	148	16,576.11	10.09
EVM0011529	*PsSWEET5*	Chr3	227	24,853.41	9.22
EVM0016366	*PsSWEET6*	Chr3	134	15,209.29	8.93
EVM0011254	*PsSWEET7*	Chr4	236	26,430.42	8.55
EVM0023285	*PsSWEET8*	Chr4	236	25,953.79	9.15
EVM0026243	*PsSWEET9*	Chr5	291	32,936.91	7.97
EVM0002608	*PsSWEET10*	Chr6	294	33,080.55	6.27
EVM0004785	*PsSWEET11*	Chr7	183	20,767.01	9.47
EVM0022638	*PsSWEET12*	Chr5	254	28,017.75	9.5
EVM0016846	*PsSWEET13*	Chr8	285	31,596.5	8.55
EVM0025419	*PsSWEET14*	Chr8	142	15,818.27	10.44
EVM0026243	*PsSWEET15*	Chr8	289	31,340.52	10.1

## Data Availability

The datasets used in this study are available from the corresponding authors upon reasonable request.

## Supplementary Materials

The following supporting information can be downloaded at https://www.mdpi.com/article/10.3390/genes14091679/s1, Table S1: Primers sequences used for RT-qPCR analysis in this study.
