# Supplementary material for: Genome-Wide Identification and Expression Profiling Analysis of SWEET Family Genes Involved in Fruit Development in Plum (Prunus salicina Lindl)
Source: Genes (Basel). 2023 Aug 25;14(9):1679. doi: 10.3390/genes14091679 (PMC10531292; doi:10.3390/genes14091679)
Supplement: Supplementary file 1 [file genes-14-01679-s001.zip › genes-2537109-supplementary.pdf]

Table S1. Primer pairs used in qRT-PCR analysis

| Gene name | Forward primer | Reverse primer |
|---|---|---|
| *PsSWEET1* | TTCTTATGTGGCACTTCATGGTTCA | ATTGATTGCTTTGATTGGTGGG |
| *PsSWEET2* | CAGTGCTCCCTCACAACTCTTTCA | GCTGTTTCAGTTTTGTTCTCGCTCA |
| *PsSWEET3* | GAGGATGCTGGGATTTCTGTTG | CAACTAAGCAGTCCAACAATCAGC |
| *PsSWEET5* | AGCTCGAAACGTCGTCGGTAT | ACAAGAAGGCTGTCTGGGTGAA |
| *PsSWEET7* | TGCCGTTGTGGTTTTCATTACTC | ACCATTTGGAAGCAGAAGGTAGG |
| *PsSWEET9* | CGGGCTCAAAACGTCAAAAGA | CGTAGACCACCCAGACAACTCCA |
| *PsSWEET10* | CGGATTAAAGTTGTTGGGTGGA | GATGTTTGGTGCCGCTATGAA |
| *PsSWEET12* | TGCCGTTTTCTTTATCCTTCTTCC | GCTGCTGCTTGTACTTGTATCTCCA |
| *PsSWEET13* | GTGGCACAGGTCATCCGAACA | GAGCAGCATCTGAAGCAACCC |
| *PsSWEET14* | TAGCTCTTAATGTAGCCTTTCCAGC | TCTTTAGTGCCACCACAGTTTTCA |
| *Actin* | AACTGGGATGACATGGAGAAGATC | AATGGCATGAGGTAGGGCATAA |
